# Multilocus sequence typing, biochemical and antibiotic resistance characterizations reveal diversity of North American strains of the honey bee pathogen *Paenibacillus larvae*

**DOI:** 10.1371/journal.pone.0176831

**Published:** 2017-05-03

**Authors:** Sasiprapa Krongdang, Jay D. Evans, Jeffery S. Pettis, Panuwan Chantawannakul

**Affiliations:** 1 Bee Protection Laboratory, Department of Biology, Faculty of Science, Chiang Mai University, Chiang Mai, Thailand; 2 USDA-ARS, Bee Research Laboratory, Beltsville, MD, United States of America; 3 Material Science Research Center, Faculty of Science, Chiang Mai University, Chiang Mai, Thailand; University of Milan, ITALY

## Abstract

*Paenibacillus larvae* is a Gram positive bacterium and the causative agent of the most widespread fatal brood disease of honey bees, American foulbrood (AFB). A total of thirty-three independent *Paenibacillus larvae* isolates from various geographical origins in North America and five reference strains were investigated for genetic diversity using multilocus sequence typing (MLST). This technique is regarded to be a powerful tool for epidemiological studies of pathogenic bacteria and is widely used in genotyping assays. For MLST, seven housekeeping gene loci, *ilvD* (dihydroxy-acid dyhydrogenase), *tri* (triosephosphate isomerase), *purH* (phospharibosyl-aminoimidazolecarboxamide), *recF* (DNA replication and repair protein), *pyrE* (orotate phosphoribosyltransferase), *sucC* (succinyl coenzyme A synthetase β subunit) and *glpF* (glycerol uptake facilitator protein) were studied and applied for primer designs. Previously, ERIC type DNA fingerprinting was applied to these same isolates and the data showed that almost all represented the ERIC I type, whereas using BOX-PCR gave an indication of more diversity. All isolates were screened for resistance to four antibiotics used by U.S. beekeepers, showing extensive resistance to tetracycline and the first records of resistance to tylosin and lincomycin. Our data highlight the intraspecies relationships of *P*. *larvae* and the potential application of MLST methods in enhancing our understanding of epidemiological relationships among bacterial isolates of different origins.

## Introduction

American foulbrood (AFB), caused by the Gram-positive bacterium *Paenibacillus larvae* (*P*. *larvae*) [1,2], is regarded as the most serious bacterial disease affecting the brood of honey bees (mainly *Apis mellifera*) worldwide [3]. *P*. *larvae* can form spores that remain viable for years and survive under extreme conditions. The bacteria can be readily transmitted by swarming, drifting, or foraging bees or by beekeepers when they exchange hive equipment and hive materials between colonies or apiaries [2,4–6]. A diseased colony can be visualized and detected only when larvae are observed with clinical symptoms of AFB, however colonies may harbor many *P*. *larvae* spores without showing symptoms [7]. In many countries, including the United States, infected honey bee colonies are treated with antibiotics when clinical AFB symptoms are present. Oxytetracycline (OTC = Terramycin^®^) is the antibiotic most often applied to treat this disease [8]. More recently, tylosin [9,10] and lincomycin [11] have been used to cure AFB [11]. In recent years, not only has AFB become more prevalent but resistance to antibiotics by *P*. *larvae* has been observed particularly oxytetracycline [12,13].

Apart from field inspection of AFB infected colonies; laboratory tests are used to confirm the presence of *P*. *larvae*. Microscopic identification of *P*. *larvae* is commonly complemented by using biochemical diagnosis and molecular techniques. Molecular typing methods are also essential in order to better understand the epidemiology of such opportunistic agents with the final goal of preventing contamination[14]. Currently, the genetic diversity of *P*. *larvae* has been studied by using various methods such as a repetitive-element PCR fingerprinting with primers ERIC [15–17], BOX-[17,18], MBO REP1, and pulsed-field gel electrophoresis (PFGE) [15,16,19,20]. Nevertheless, these methods are difficult to standardize between laboratories especially with large collection of bacterial strains. Therefore, standardized approach would greatly help us to study population structure and evolution of the *P*. *larvae*. Multilocus sequence typing (MLST) has now become standard for assessing genetic variation in worldwide collections of bacterial isolates. MLST schemes discriminate several bacterial and yeast species at the strain level by using information from across multiple independent markers [21]. In addition, the level of genomic plasticity of *P*. *larvae* has not been clarified, this highlights the need for a new robust tools for strain differentiation using phylogenetic analysis [22]. In this study, we designed an MLST scheme for the *P*. *larvae* from infected colonies from different geographical organism in United States. We used non-overlapping loci and our research was done in parallel to and similar to the approach recently invoked for European strains of this species by Morrissey et al., 2015 [23]. We compared sequence data against traits important for the evolution, phylogeny, and epidemiology of these bacteria, including biochemistry profiles and antibiotic susceptibility.

## Materials and methods

### Bacterial isolates

**Fig 1:**
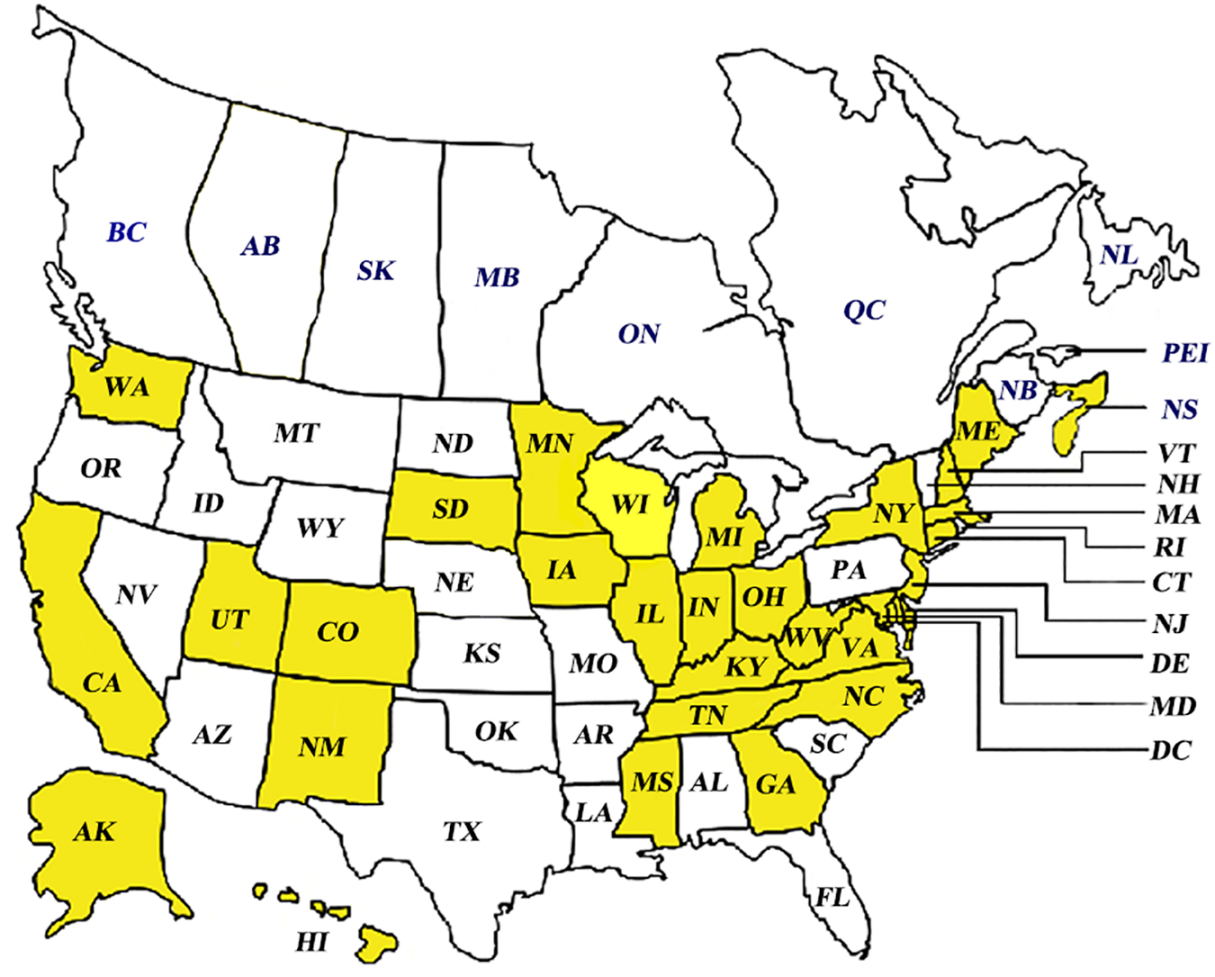
Different geographical AFB sample origins across North America (yellow shading indicates *Paenibacillus larvae* collection areas). For more information on sample location and or identification please see S1 Table.

A total of 33 field isolates of *Paenibacillus larvae* were sampled from those received by the Bee Disease Diagnostic Service (USDA-ARS, MD, US) from different regions in the United States in the years 1999–2013 (Fig 1). These isolates were contrasted with *P*. *larvae* reference strains ATCC9545, ATCC49843, 233/00, LMG16247 and LMG16252 (described in S1 Table). Honey bee larvae with AFB symptoms were dissolved in 200 μl sterile distilled water and incubated at 80°C for 15 min in order to kill vegetative cells of bacteria, yeasts and non-spore forming bacteria before culturing [18]. The isolates were cultivated using THClYGP agar plates [15] using the following recipe (per liter): 15 g yeast extract (Fisher Science), 1 g pyruvic acid (EMBSCO), 200 ml 0.1 M Tris-HCl, pH 7.0, 20 g agar, 40 ml 10% glucose and 0.6 ml nalidixic acid. Plates were incubated at 37°C for 3 days.

### Primer design for MLST

Thirteen housekeeping loci were chosen for the MLST primer design using the following criteria: presence as a single copy in all strains, rate of sequence conservation and wide distribution across the chromosome. *Bacillus cereus* and *Bacillus licheniformis* MLST databases were used as templates from PubMLST (http://pubmlst.org). Full-length sequences of seven genes were identified from the *Paenibacillus larvae* DSM 25430 genome assembly (NC_023134.1). Nucleotide sequences were downloaded from GenBank and aligned with template allele profiles and analyzed by using the BLAST and FASTA software available at the National Center for Biotechnology website (http://www.ncbi.nlm.nih.gov). Specific primers of each gene were designed from the most conserved regions using Primer3 and BLAST software (http://www.ncbi.nlm.nih.gov/tools/primer-blast/) within suitable length between 400–700 bp and similar % GC and annealing temperatures.

### DNA extraction and DNA fingerprinting by using ERIC- and BOX PCR

Preparations of bacterial DNA for DNA fingerprinting and MLST analysis were performed as described by Genersch and Otten [19]. A total genomic DNA was isolated from *P*. *larvae* colonies grown on culture plates using a 6% InstaGene matrix (Bio-Rad, Hercules, CA) following the manufacturer’s instructions. PCR amplification was performed using primer ERIC1R (5’-ATGTAAGCTCCTGGGGATTCAC- 3’) and ERIC2 (5’-AAGTAAGTGACTGGGGTGAGCG-3’)[24] for ERIC gene and BOX A1R (5’-CTACGGCAAGGCGACGCTGACG-3’) for BOX gene [24, 25]. PCR were carried out in a final volume 50 μl which consisted of 1X PCR buffer (containing 1.5 mM MgCl_2_); 1x Q-Solution (for ERIC amplification); deoxynucleoside triphosphate mix (dGTP, dTTP, dATP and dCTP, each at 2 mM); 0.2 μM of each primer; 2.5 U HotStarTaq DNA polymerase (QIAGEN, Germantown, MD) and 10 ng of DNA. The thermal cycle program consisted of initial activation step (15 min, 95°C), followed by 35 cycles of denaturation at 94°C for 1 min, annealing for 1 min at 53°C and extension for 2.5 min at 72°C, followed by a final elongation step at 72°C for 10 min. 5 μl of the ERIC and the BOX-PCR products were analyzed in 1.5% agarose gel, stained with ethidium bromide, visualized and photographed under UV light.

### MLST PCR amplification

For MLST, PCR reactions were carried out in a final volume of 30 μl containing 10 ng of DNA and 0.2 μM of each primer along with the PCR reactions described above. Cycling conditions included an initial denaturing step of 95°C for 15 min, 35 cycles at 94°C for 30 sec, 55°C for 45 sec, and 72°C for 1 min, and a final extension step at 72°C for 10 min. Amplification products were resolved electrophoretically by 1.5% agarose gel, stained with ethidium bromide and photographed under UV transillumination to confirm an expected size and single clear band per PCR. Then, PCR products were purified directly using QIAquick PCR purification kit (QIAGEN, Germantown, MD) following the manufacturer’s instructions. The sequencing was performed according to Big Dye 2.0 (Applied Biosystems, Carlsbad, CA) according to the manufacturer’s instructions. DNA sequences obtained in this study were submitted to GenBank.

### MLST and Phylogenetic analysis

Sequences from all loci were concatenated prior to analysis. An alignment of the sequences obtained together with reference sequences was constructed for each of genetic markers using the program MEGA6 [26]. Neighbor joining trees were constructed using the program MEGA6, based on the evolutionary distances calculated by the Kimura-2-parameter model. The reliability of these trees was assessed using bootstrap analyses across 1000 replicates. DNA polymorphism was calculated in DnaSP 5.10.01 [27]. Number of haplotypes (Hap) and haplotype diversity (Hd) were calculated. The nucleotide diversity per site (θ) and the average number of nucleotide difference between populations (π) were determined as well. The neutrality of mutation was analyzed by Tajima’s D [28], Fu and Li’s D*, Fu and Li F* [29] and Fu’s Fs [30] selective neutrality tests. A unique number was assigned to each distinct allele sequence for each locus. Each unique combination of seven loci numbers was determined the sequence type (ST) of each strain based on the analysis of nucleotide polymorphisms. The same ST was used for isolates sharing the same allelic profiles and calculated for relatedness using the eBURST v3 program [31].

### Phenotypic characterizations of *P*. *larvae*

The field isolates were characterized biochemically using the commercially available API 50 CHB, API ZYM and API 20E systems (bioMérieux, Boston, MA). The tests were carried out according to the manufacturer’s instructions with minor modifications as follows: *P*. *larvae* colonies from THClYGP agar were suspended in 3 ml sterile saline solution in order to obtain a turbidity of 5–6 on the McFarland scale. Each well was filled with three to four drops of the culture suspension. The dendrogram obtained from biochemistry profile was constructed by the UPGMA method applying Pearson’s correlation.

### Antibiotic susceptibility testing

Antimicrobial susceptibility tests were assessed by the disk diffusion method. Four antibiotic drugs; OTC (30 μg/disk), tetracycline (5 μg/disk), tylosin (30 μg/disk) and lincomycin (2 μg/disk) were examined. *P*. *larvae* strains were cultured on the agar MYPGP for 48 h of incubation at 37°C were suspended in sterile distilled water and adjusted to approximately 2.87 × 10^8^ cells/ml (A620 nm = 0.388, equivalent to a McFarland standard no. 1[10]. Diameters of the inhibition zones were measured. Three replicates were tested for each isolate strain.

## Results

### Genotyping by ERIC- and BOX PCR analysis

ERIC PCR is generally used to characterize *P*. *larvae*. In comparison to reference strains which described ERIC types as ATCC 9545I (ERIC I), 233/00 (ERIC II), LMG16252 (ERIC III), ERIC ATCC 49843 and LMG 16247 (ERIC IV), all *P*. *larvae* isolates were clustered together and showing the only one of distinguishable genotypes, in ERIC I genotype with fragments ranging from 200 to 3000 bp (S1 Fig).

**Fig 2:**
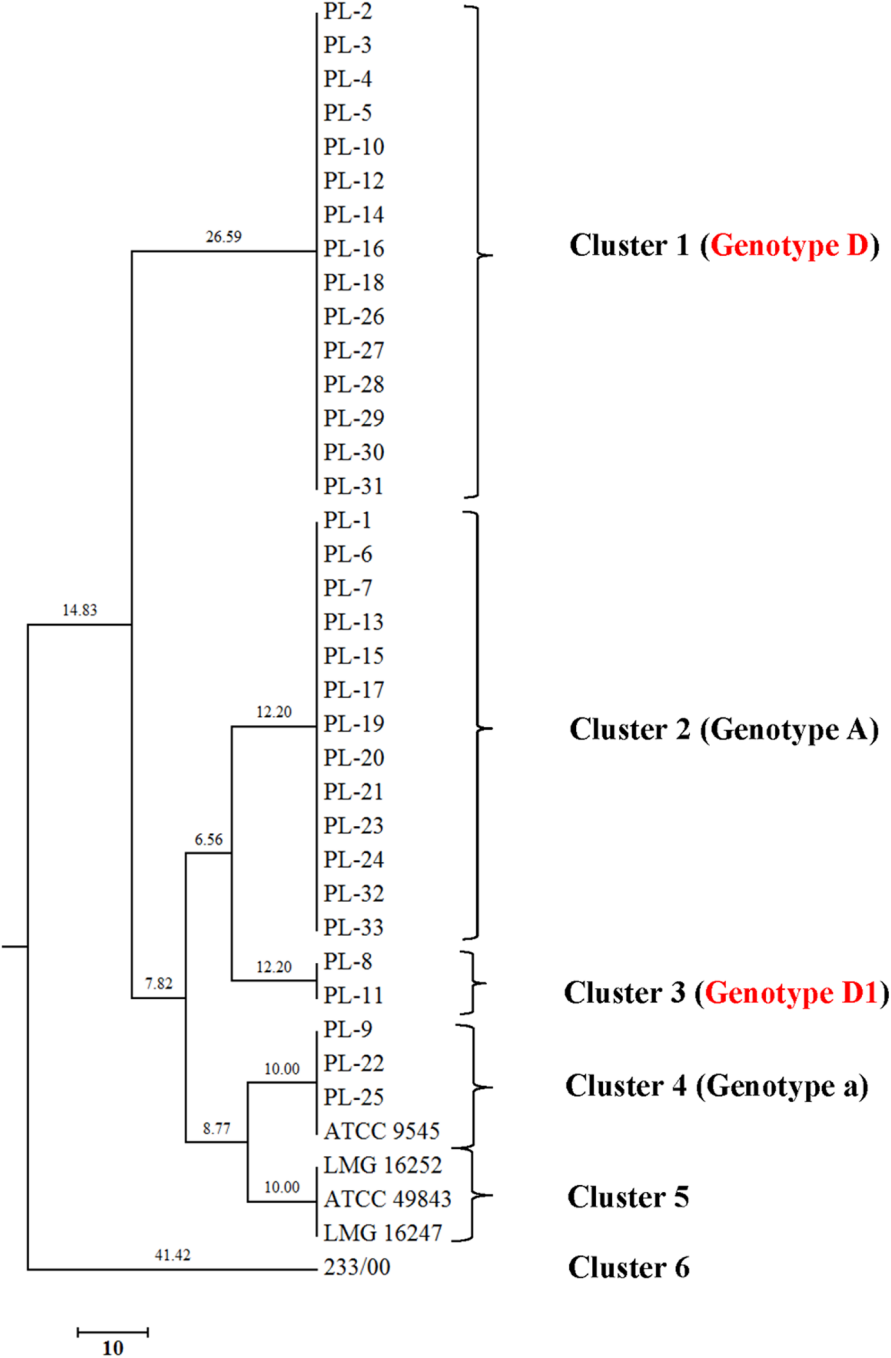
Dendrogram showing the UPGMA cluster analysis of BOX-PCR profiles generated using BOX1R primer (Pearson correlation) of 38 *Paenibacillus larvae* isolates.

Genomic fingerprints of *P*. *larvae* isolates also were found by using BOX and the resulting dendrogram revealed UPGMA cluster with six different clusters (Fig 2). Two of the total which are named genotype BOX A and BOX B corresponded to genotypes described by Alippi and Marioaguilar [20]. Additionally, Genersch and Otten [19] and Loncaric et al. [17] who labeled these as genotype a and A. Interestingly, new genotypes were observed and given names as BOX D and BOX D1. The new genotypes present two band sizes around 200 to 300 bp, which were absent in the others. BOX D and BOX D1 showed highly similar patterns and bands around 200–300 bp which were missing in others. The band size 200 bp can be observed in BOX D1 and the reference strain 233/00. Hence, as expected BOX-PCR provided a better discrimination than ERIC-PCR, because all isolates had been included in ERIC I genotype (S2 Fig).

### Sequence polymorphism and multilocus genotypes

Thirteen housekeeping gene were successfully amplified and tested for nucleotide diversity in 33 isolates and 5 reference strains (S3 Fig). Seven housekeeping gene loci, *ilvD* (dihydroxy-acid dyhydrogenase), *tri* (triosephosphate isomerase), *purH* (phospharibosyl-aminoimidazolecarboxamide), *recF* (DNA replication and repair protein), *pyrE* (orotate phosphoribosyltransferase), *SucC* (succinyl coenzyme A synthetase β subunit) and *glpF* (glycerol uptake facilitator protein) were chosen for the MLST analysis (Table 1) and others showed no diversity within the sequence (S2 Table). All sequences have been deposited in GenBank (https://www.ncbi.nlm.nih.gov/genbank/) under accession numbers from: KY673263-KY673300 for *ilvD*, KY673301-KY673338 for *purH*, KY673339-KY673376 for *tri*, KY673377-KY673414 for *recF*, KY673415-KY673452 for *pyrE*, KY673453- KY673490 for s*ucC* and KY673491-KY673528 for *glpK*.

**Table 1:** New oligonucleotide primer sequences and descriptions for the amplification and sequencing of seven MLST loci for *Paenibacillus larvae*.

Primers	Sequence (5'->3')	Gene product	Product length (bp)	Tm (°C)	%GC
ilvD_F	ATAAGGACG TCATTCGCCCA	Dihydroxy-acid dehydratase	577	59.17	50.0
ilvD_R	GTGCTCGCCGATGTAACAAG			59.63	55.0
purH_F	CGGCAGATTACGGCAAAGTG	Phospharibosylaminoimidazolecarboxamide	458	59.90	55.0
purH_R	GGTAGGGTCGGCTTCATACG			60.39	60.0
tpi_F	CCGGAGAAATCAGC GTATCA	Triosephosphate isomerase	540	59.93	52.4
tpi_R	GGACATCTTACT TCGCCCCC			60.04	60.0
sucC_F	TCGTACATATGCGGGTGAGC	Succinyl-CoA ligase [ADP-forming] subunit beta	656	59.97	55.0
sucC_R	CAAGGAAGTTGGCCGGATCT			60.04	55.0
pyrE_F	TTCATCAGAGAGCGGGAACA	Orotate phosphoribosyltransferase	659	58.73	50.0
pyrE_R	TCCGCCAAGCCTGTAATTGT			59.96	50.0
recF_F	TGAACAGATTCACGACGGCA	DNA replication and repair protein	555	59.97	50.0
recF_R	TGAGCTCGGAAGACCGGATA			60.11	55.0
glpK_F	AGTCTGCTTCCATGGCCT TC	Glycerol kinase GlpK	762	60.4	55.0
glpK_R	TGGATCATGTGGAAGGTGCC			60.3	55.0

On average, housekeeping genes had a low level of nucleotide (π = 0.00086±0.00017) and haplotype diversity (Hd = 0.19885±0.07585) in *P*. *larva*e isolates. *glpK* had the highest sequence polymorphism and revealed the presence of 6 genotypes with an Hd of 0.328. Whereas *tri* locus showed the lowest sequence polymorphism, with two distinct genotypes, and an Hd of 0.149 (Table 2). The number of alleles and polymorphic sites identified per locus was overall low. This was reflected in the low θ and π values calculated for each gene. The G+C mol content varied between 0.4407 for the partial *recF* sequence to 0.5077 for the *ilvD* sequence. By Tajima’s D, Fu and Li’s D, Fu and Li’s F test, two of the housekeeping genes revealed signs of significant deviation from neutrality (Table 2); however, Fu and Fs’ D test did not show significant. Therefore, these tests suggest that these housekeeping genes are primarily evolving in a neutral fashion and can be used as genetic markers for inferring population and evolutionary history of *P*. *larvae*.

**Table 2:** Sequence data obtained from sequencing for seven housekeeping genes of *Paenibacillus larvae* in this study.

Loci	Sites of analysis (bp)^a^	S^b^	Hap^b^	Hd^b^	G+C content	π^b^	θ^b^	k^b^	Neutrality test			
									TajimaD^c^	FuLiD^c^	FuLiF^c^	FuFs^c^
*ilvD1*	486	3	2	0.153	0.5077	0.00095	0.00148	0.4595	-0.8019	0.9246	0.4847	1.592
*PurH*	355	5	4	0.162	0.4805	0.00078	0.00341	0.2778	-2.0071*	-3.4154**	-3.4863**	-2.433
*Tri*	437	3	2	0.149	0.4744	0.00103	0.00164	0.4481	-0.8225	0.9215	0.4738	1.557
*recF*	460	6	5	0.158	0.4407	0.00094	0.0032	0.4234	-1.9154*	-2.8568*	-3.0006*	-1.428
*pyrE*	565	2	2	0.153	0.4829	0.00054	0.00085	0.3063	-0.6955	0.7802	0.4111	0.859
*sucC*	570	4	4	0.289	0.4477	0.00095	0.00167	0.542	-1.0411	-0.0235	-0.3819	-0.842
*glpK*	675	5	6	0.328	0.493	0.00093	0.00175	0.6262	-1.2043	-0.725	-1.0162	-2.795

^a^ Size of fragments after alignment and cutting.

^b^ S, number of polymorphic sites; Hap, number of haplotypes; Hd, haplotype diversity; **π**, nucleotide diversity per site; θ, average number of nucleotide difference per site; k, average number of nucleotide differences.

^c^ Statistical analysis of neutrality tests.TajimaD, Tajima’s D test; FuLiD, Fu and Li’s D test; FuLiF, Fu and Li’s F test; FuFs, Fu’s Fs test; Statistically significant was indicated by using

* (P <0.05) and

** (P < 0.02).

**Fig 3:**
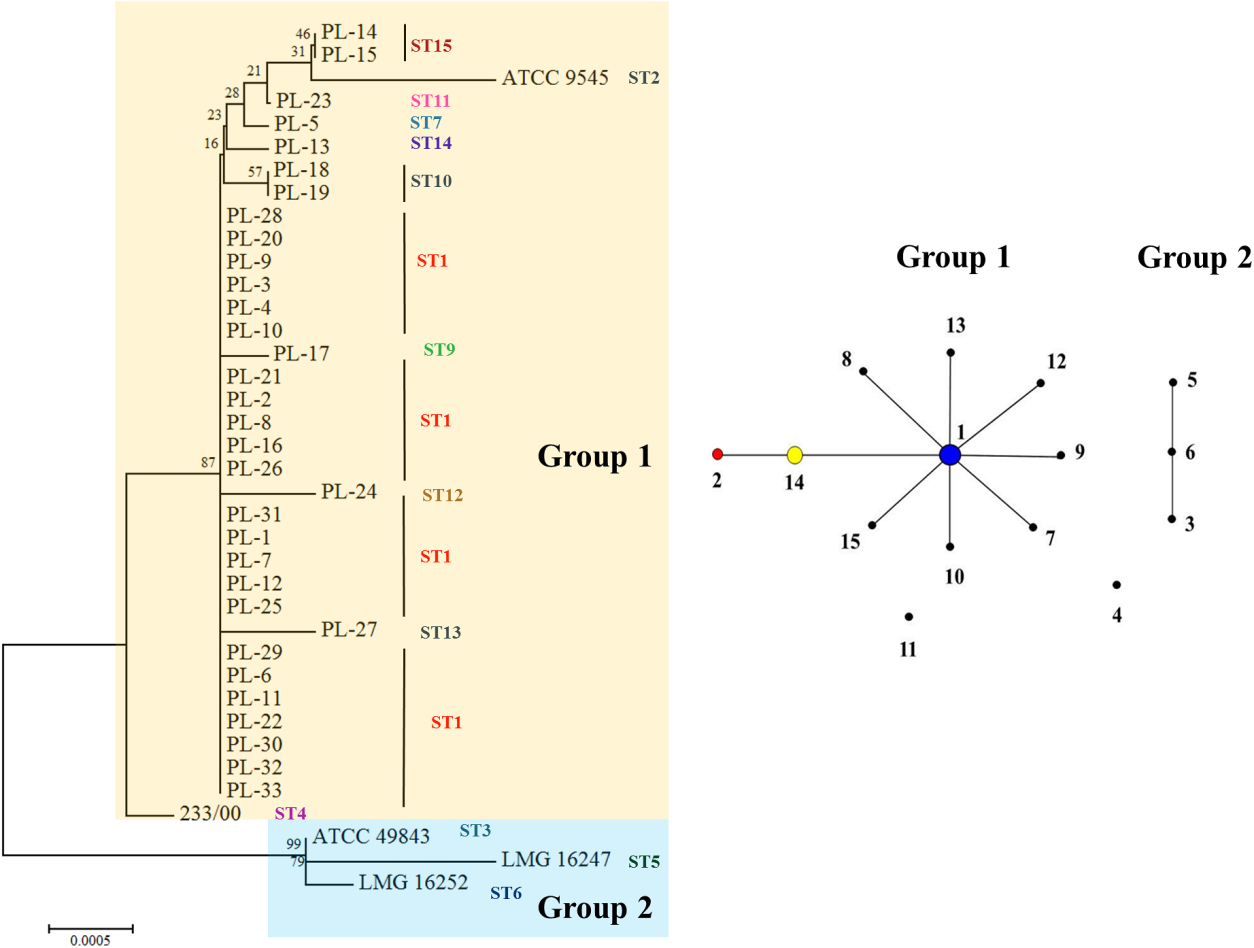
Presentations of MLST schemes. (A) MLST Phylogenetic tree (neighbor-joining) of 38 *Paenibacillus larvae* isolates after concatenating the sequences of seven loci. The phylogenetic tree was constructed using the neighbor-joining method with 1,000 bootstraps (bootstrap values are shown above the nodes). Phylogenetic analyzes were constructed in MEGA6. (B) eBURST analysis of MLST profiles of 15 STs of *Paenibacillus larvae* isolates.

MLST assays showed a high degree of evolutionary divergence between North American *P*. *larvae* isolates. The neighbor-joining tree constructed using the concatenated sequences of the seven housekeeping loci identified major branches with acceptable (50%) bootstrap values. Two main genetically isolated groups were observed (Fig 3A). Group 1 consisted of 35 isolates and group 2 contained 3 isolates. The data in the eBURST analysis showed 15 different sequence types (Table 3). All STs could be grouped into 2 groups. Ten STs which belonged in group 1 are ST1, ST2, ST3, ST7, ST8, ST9, ST10, ST12, ST13, ST14 and ST15 which ST1 were identified as founder. Three STs are belonged to group II (ST3, ST5 and ST6). All most *P*. *larvae* from United States were classified into a distinct clade (Group 1) while reference isolates that identified in ERIC type III and IV were grouped into another clade (Group 2). The other 2 STs that did not belong to any groups were identified as singletons (ST4 and ST11) (Fig 3B).

**Table 3:** Genotypes of the 38 *Paenibacillus larvae* isolates tested.

Strains	ST	Housekeeping genes allele number						
		*ilvD1*	*purH*	*tri*	*recF*	*pyrE1*	*sucC*	*glpK*
PL-1, PL-2, PL-3, PL-4, PL-5, PL-6, PL-8, PL-9, PL-10, PL-11, PL-12, PL-16, PL-20, PL-21, PL-22, PL-25, PL-26,PL-28, PL-29, PL-30, PL-31, PL-32, PL-33	1	1	1	1	1	1	1	1
ATCC 9545	2	1	1	1	1	1	4	2
ATCC 49843	3	2	2	2	2	2	2	1
233/00	4	1	2	1	3	1	3	1
LMG 16247	5	2	2	2	4	2	2	1
LMG 16252	6	2	2	2	5	2	2	1
PL-5	7	1	1	1	1	1	1	3
PL-7	8	1	2	1	1	1	1	1
PL-17,	9	1	3	1	1	1	1	1
PL-18, PL-19	10	1	1	1	1	1	1	5
PL-23	11	1	1	1	1	1	1	6
PL-24	12	1	4	1	1	1	1	1
PL-27	13	1	5	1	1	1	1	1
PL-13	14	1	1	1	1	1	4	1
PL-14, PL-15	15	1	1	1	1	1	1	4

### Antibiotic susceptibility

**Fig 4:**
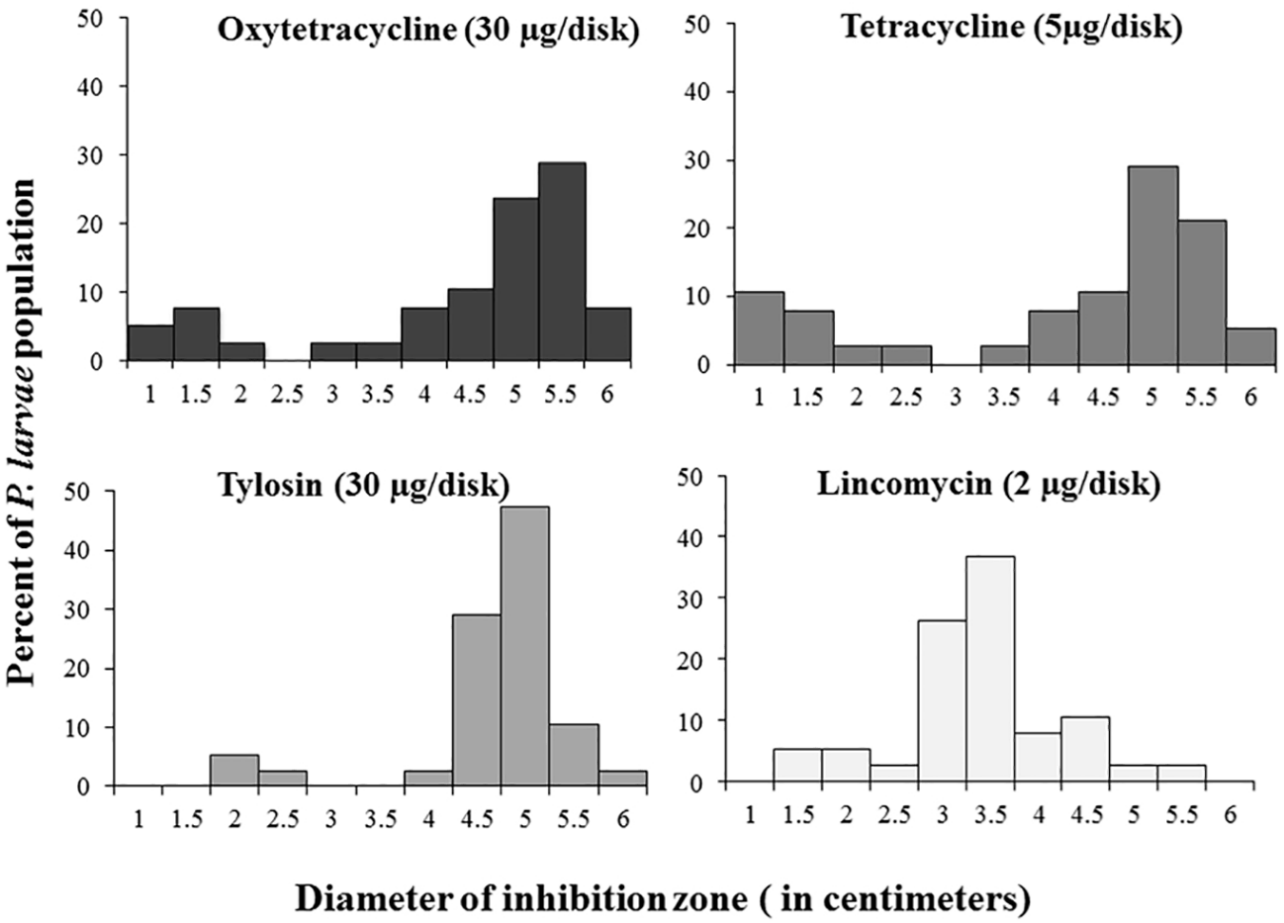
Histogram showing the susceptibility of *Paenibacillus larvae* isolates to four antibiotics.

Susceptibility of antibiotics was examined by using antibiotic disks containing 30 μg oxytetracycline (OTC), 5 μg tetracycline, 30 μg tylosin and 2 μg lincomycin. A histogram plot shows contributions of clear zone diameters in each antibiotics group (Fig 4).

**Fig 5:**
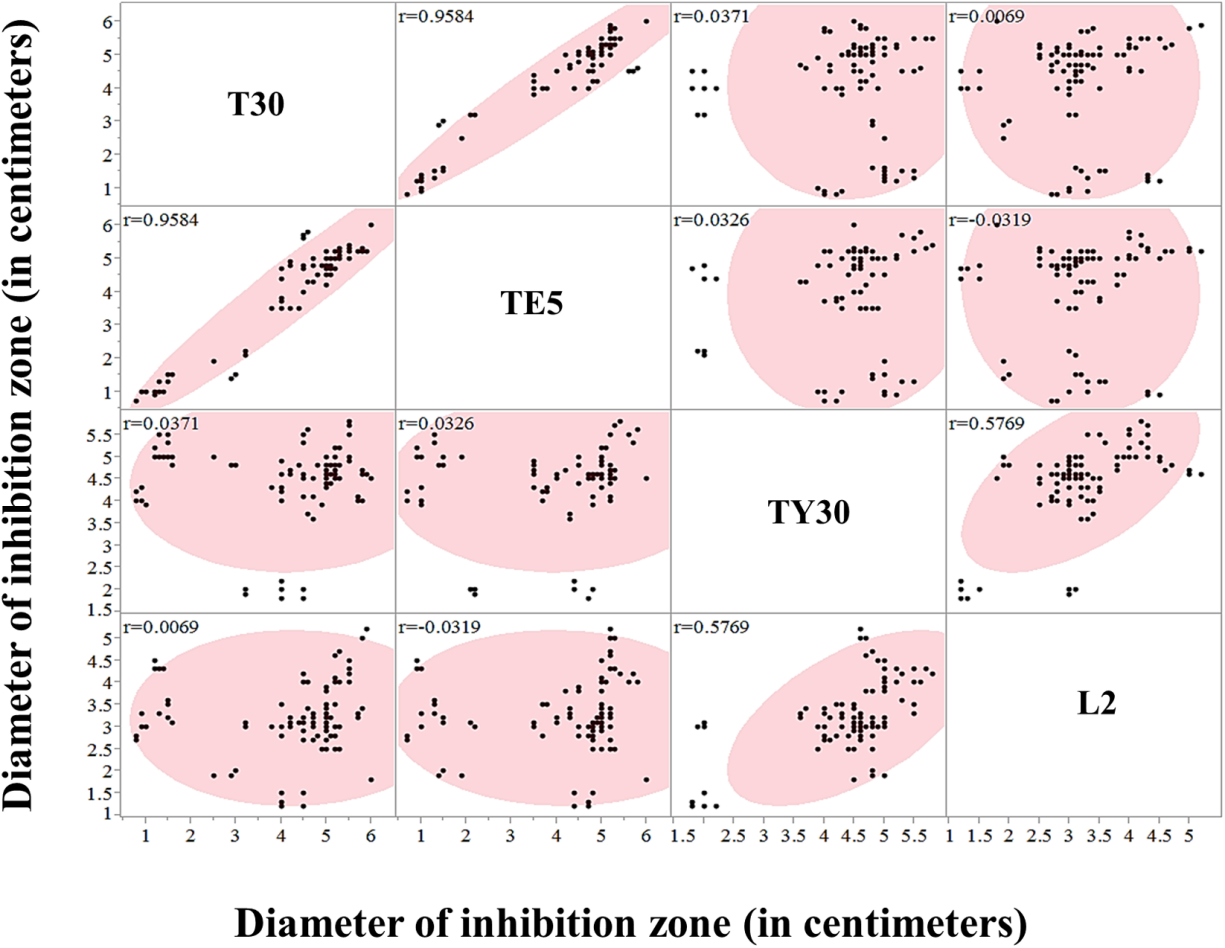
Correlations of four antibiotic groups. Diameter of inhibition zone of four antibiotics were scattered and correlation (r^2^) in each two antibiotics were shown.

The histogram showed that susceptibility toward antibiotics of *P*. *larvae* isolates has similar trends. The plot showed a clear split between resistant and susceptible isolates with a defined zone of inhibition difference of 2.0 cm between resistant and susceptible isolates. In this work, OTC, tetracycline, tylosin and lincomycin-resistant isolates were found as 15.78% (PL-3, PL-4, PL-5, PL-15, PL-29, and PL-30), 21.05% (PL-3, PL-4, PL-5, PL-9, PL-15, PL-28, PL-29, and PL-30), 7.8% (PL-16, PL-18 and PL-26) and 7.8% (PL-18, PL-26 and ATCC 9545) of our screened isolates, respectively. Susceptibility of two antibiotics, OTC and tetracycline were tightly correlated (R^2^ = 0.8706) as expected due to their similar mode of action and molecular structure. In addition, resistance to tylosin and lincomycin (R^2^ = 0.5757) tended to occur in identical isolates, despite different modes of action for these compounds. No further correlations across isolates for antibiotic susceptibility between treatments were found (Fig 5).

### Biochemical characteristics of *P*. *larvae*

**Fig 6:**
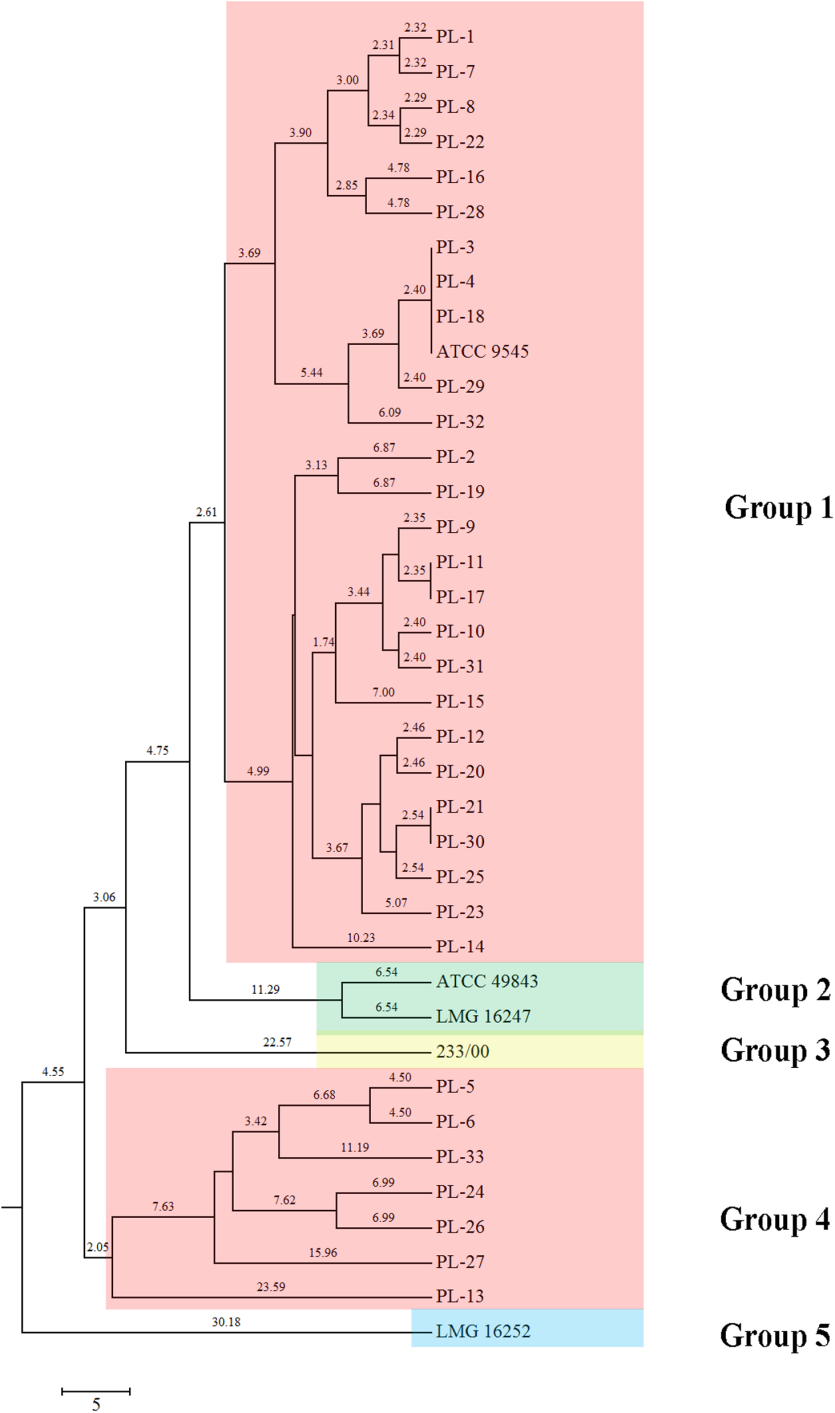
UPGMA dendrogram of *Paenibacillus larvae* biochemistry by using API^®^ systems.

Carbohydrate utilization was characterized by using API^®^ 50CH system with 49 carbohydrates (S3 Table). In terms of metabolic differences of *P*. *larvae* isolates, the dendrogram was constructed by using biochemistry profiles. Five different metabolic profiles were observed by using UPGMA method. The isolates defined as ERIC I mostly belong to group 1 and 4 whereas, the reference strains, ATCC 49843 and LMG 16247 (ERIC IV), 233/00 (ERIC II), and LMG 16252 (ERIC III) were clearly related with ERIC DNA fingerprinting (Fig 6).

All *P*. *larvae* hydrolyzed carbon sources such as esculin ferric citrate and potassium-2-ketogluconate and phosphorus sources effectively. 97.39% utilized N-acetylglucosamine and D-trehalose (excluding PL-13) and 94.74% (36/38) used glycerol (excluding PL-13, LMG 16252), D-ribose (excluding PL-13, PL-15). Abundant hydrolytic enzyme productions were detected during inoculates time with API^®^ZYM. The results showed all *P*. *larvae* isolates produce esterase (C4), esterase lipase (C8) acid phosphatase, naphthol-AS-BI-phosphohydrolase and N-acetyl-β-glucosaminidase. 97.39% produced alkaline phosphatase (excluding isolate 233/00). Using the API^®^20E system, all isolates showed the positive reaction for utilizing of gelatin and sodium pyruvate.

## Discussion

Recently, new *P*. *larvae* bacterial isolates have been reported in Austria [17] and Egypt [32]. These studies have used the molecular strategies of genotyping, biochemistry profiles or/and antibiotic susceptibility to generally investigate bacterial genetic diversity, but those methods did not improve *P*. *larvae* intraspecific discrimination [16]. In the current research, our aim was to apply multilocus sequence typing (MLST), as an improved approach to explore genetic variability and population dynamics. Development of an MLST scheme has been done in several bacterial groups such as *Bacillus cereus* [21], *Bacillus thuringiensis* [33] and *Clostridium botulinum* [34], and most recently in an independent effort conducted in primarily European isolates of *P*. *larvae* [23]. This study represents the first report of a broad characterization of *P*. *larvae* in North America, and doubles the set of candidate loci for applying MLST approaches for this key species. The population genetics of *P*. *larvae* were examined by multilocus sequence typing of 38 isolates by concatenating sequences of all seven polymorphic markers (*ilvD*, *purH*, *tri*, *recF*, *pyeE*, *sucC* and *glpK*). Here, 15 new STs were analyzed and presented by eBURST. Typically, field isolates are ST1. The others we found were rare alleles present at low frequencies (ST2-ST15). Two distinct groups were identified in the sampled population. Group 1 contained isolates from North America whereas in Group 2 reflected the reference strains obtained from European isolates (ATCC49843, LMG 16247 and LMG 16252) [15]. ST4 (233/00) and ST11 (PL-23) were singletons in this study. Our ST4 isolate derived from Sweden [15] while ST11 was obtained from Alaska (USA), suggesting geographic distinctiveness in these regions. Overall, the results discriminated the strains with high resolution and showed genetic structure across different geographical regions. This is similar to geographic separation by Margos et al. [35] using MLST analysis of the bacterium *Borrelia burgdorferi* in North American and European populations. Their study demonstrated that the concatenated sequences of *B*. *burgdorferi* housekeeping genes corresponded to different geographical regions. Morissey et al., 2015 [23] reported an MLST analysis of *P*. *larvae* that showed ability of this method to identify relationships between geographic distance and genetic distance amongst native and non-native countries (mostly, in Europe) and found that isolated typed as ERIC I were more diverse than others. Similarly, our reports showed more diversity in ERIC I. However, they also suggested that the ERIC II type is broadly distributed a result we did not find. Generally, *P*. *larvae* populations showed relatively little polymorphism. Two loci, *purH* and *recF*, showed significant negative values by Tajima’s test, indicating purifying selection or population expansion of these genes [28]. The others appear to be neutral housekeeping genes useful for inferring the evolutionary history of *P*. *larvae* in North America. It could be implied that *ilvD1*, *tri*, *pyre*, *sucC* and *glpK* are especially free from directed selection pressure, and therefore will be most useful for understanding demographic events and bacterial population history [36].

Antibiotic susceptibility may also be useful for epidemiology studies and lead to the detection of resistant strains [8]. Historically the AFB disease has been controlled either by the destruction of infected colonies (burning), or by antibiotic use. For instance, OTC and tetracycline are the same class of antibiotics. OTC is commonly used for AFB control, although resistant strains have been reported in several countries [13]. Tylosin and lincomycin are known to be effective against resistant AFB strains in the field in both Florida and New Jersey [8]. Tylosin is non-toxic to honey bees and is also used to control AFB [37]. In an earlier study, Alippi et al., 2005 [10] did not detect resistance by *P*. *larvae* toward tylosin. Likewise, lincomycin is a third alternative used to inhibit the growth of OTC resistant *P*. *larvae* [8]. Hence, in this study, we provide the first report of isolates of *P*. *larvae* showing resistance to tylosin and lincomycin. However, OTC remains the only approved drug treatment available in the United States for the prevention and control of AFB [4]. Interestingly, All OTC-resistant isolates were defined to ERIC I-BOX D genotype. However, OTC-resistant trait was not correlated with bacterial haplotype. This might be due to either resistance that has evolved multiple times in *P*. *larvae* or resistance caused by recent horizontal transfer via a non-genomic route (e.g. plasmids or conjugal transposons) [12]. In an earlier study, Alippi et al., 2005 [10] did not find tylosin resistance in this species. Hence, here we provide the first report of isolates of *P*. *larvae* showing resistance to tylosin and lincomycin.

Phenotypic comparisons using three API^®^ system analyses of the *P*. *larvae* strains were quite contradictory and did not relate with sequence types. However, our results correspond with Neuendorf et al., 2004 [38]. In contrast, Kilwinski et al., 2004 [39] reported mannitol is solely accepted as a metabolic source by *P*. *larvae*. Our results are quite similar because all the field isolates reacted negatively for mannitol while the reference strains (ATCC 49843, LMG16247, LMG16252 and 233/00) can utilize this carbohydrate.

Analyses across the biochemical, phylogenetic, and molecular typing methods highlighted relatively high intraspecific diversity across this *P*. *larvae* collection and indicated fairly consistent grouping of isolates across all three methods. Phenetic analysis had previously been grouped and compared with genotype. Nevertheless, it seems that biochemical typing provides less discrimination than DNA fingerprinting [18]. Hamdi et al., 2013[40] suggested that relatively high intra-species molecular and phenotypic diversity did not differ significantly with *in vivo* virulence of *P*. *larvae* genotypes. This reflects that the genotypic/phenotypic differences are neutral or more related to ecological aspects that pathogenicity or virulence.

In conclusion, we propose the application of these seven loci for an MLST scheme for *P*. *larvae*, arguably in conjunction with those described by Morissey et al., 2015 [23]. This scheme showed higher discrimination than ERIC and BOX DNA fingerprints, whereas biochemistry test tended to be more variable among different strains studied. The observation of antibiotic susceptibility suggests the presence of new ST and antibiotic drug resistance in a limited number of *P*. *larvae* strains in North America. The data presented here using the MLST scheme strongly supports this technique as a powerful novel method for studying *P*. *larvae* population genetics and epidemiology.

## Supporting information

S1 Table Origins and typing data of *Paenibacillus larvae* strains analyzed in this study (1999–2013). (PDF)

S2 Table Biochemistry profiles of *Paenibacillus larvae* by using API^®^ tests. (PDF)

S3 Table Other MLST primers of *Paenibacillus larvae* were tested and showed no diversitywithin the target sequences. (PDF)

S1 Fig ERIC PCR genotyping of 38 *Paenibacillus larvae* isolates. (PDF)

S2 Fig BOX PCR genotyping of 38 *Paenibacillus larvae* isolates. (PDF)

S3 Fig Presentation of all amplified products by using designed MLST primers. In red boxes are seven loci used in the studies.
(PDF)
