# Supplementary material for: Multilocus sequence typing, biochemical and antibiotic resistance characterizations reveal diversity of North American strains of the honey bee pathogen Paenibacillus larvae
Source: PLoS One. 2017 May 3;12(5):e0176831. doi: 10.1371/journal.pone.0176831 (PMC5415181; doi:10.1371/journal.pone.0176831)
Supplement: S1 Table — (PDF) [file pone.0176831.s001.pdf]

S1 Table. Origins and typing data of *Paenibacillus larvae* strains analyzed in this study (1999-2013).

No	USDA Code#	geographical origin	Sources	Year	ST	ERIC
1	BRL230035	MD	Scales	2003	1	I
2	BRL2011-30	MI	Scales	2011	1	I
3	BRL230282	SD	Scales	2003	1	I
4	BRL230042	MS	Scales	2003	1	I
5	BRL230006	MN	Scales	2003	7	I
6	BRL280163	UT	Scales	2008	1	I
7	BRL250016	NJ	Scales	2005	8	I
8	BRL240041	WI	Scales	2004	1	I
9	BRL260037	IL	Scales	2006	1	I
10	BRL270100	WV	Scales	2007	1	I
11	BRL270081	NS	Scales	2007	1	I
12	BRL260562	CT	Scales	2006	1	I
13	BRL991122	CA	Scales	1999	14	I
14	BRL991121	TN	Scales	1999	15	I
15	BRL270005	NS	Scales	2007	15	I
16	BRL5137	GA	Scales	2013	1	I
17	BRL260120	NY	Scales	2006	9	I
18	BRL270044	IN	Scales	2007	10	I
19	BRL270406	CO	Scales	2007	10	I
20	BRL270006	NM	Scales	2007	1	I
21	BRL260108	KY	Scales	2006	1	I
22	BRL270015	NS	Scales	2007	1	I
23	BRL250057	AK	Scales	2005	11	I
24	BRL270269	NC	Scales	2007	12	I
25	BRL280079	OH	Scales	2008	1	I
26	BRL232	IA	Scales	2010	1	I
27	BRL230041	VA	Scales	2003	13	I
28	BRL991123	WA	Scales	1999	1	I
29	BRL280268	IA	Scales	2008	1	I
30	BRL250031	ME	Scales	2005	1	I
31	BRL270007	MI	Scales	2007	1	I
32	BRL5136	NY	Scales	2013	1	I
33	BL2524	HI	Scales	2013	1	I
34	ATCC9545[†]		Received as a culture		2	I
35	ATCC49843[†]		Received as a culture		3	IV
36	LMG16247[†]		Received as a culture		5	IV
37	LMG16252[†]		Received as a culture		6	III
38	233/00		Received as a culture		4	II

[†] Reference strains
