# Supplementary material for: Multilocus sequence typing, biochemical and antibiotic resistance characterizations reveal diversity of North American strains of the honey bee pathogen Paenibacillus larvae
Source: PLoS One. 2017 May 3;12(5):e0176831. doi: 10.1371/journal.pone.0176831 (PMC5415181; doi:10.1371/journal.pone.0176831)
Supplement: S2 Table — (PDF) [file pone.0176831.s002.pdf]

S2 Table. Other MLST primers of *Paenibacillus larvae* were tested and showed no diversity within the target sequences.

Primers	Sequence (5'->3')	Gene product	Product length (bp)	Tm (°C)	%GC
rpoB_F	CTTTGCCTCATACGGGCCAT	rpoB DNA-directed RNA polymerase subunit beta	692	60.47	55.00
rpoB_R	TCTCCGCATCCGTACCAAAC			60.11	55.00
adk_F	TGCAGGCAAAGGTACACAGG	Adenylate kinase	564	60.54	55.00
adk_R	TCACCGTTAACCTCTCGCAG			59.76	55.00
gmk_F	TAGCCCACCGACTTTCCAAC	Guanylate kinase putative	583	59.96	55.00
gmk_R	AGGCATTGCGGACTTCATCA			60.04	50.00
aroE_F	TCTGGG GCTGCCTGTATTTG	Shikimate dehydrogenase AroE	370	60.03	55.00
aroE_R	GAACCGGAGCTTTTTGGCAG			60.04	55.00
arcC_F	AGCAGACAGTACAGCGACAC	carbamate kinase ArcC	742	60.04	55.00
arcC_R	TGTAATGATGGTGCCGGCTT			60.03	55.00
infB_F	CGTGATATCGGTGACCTGGG	Translation initiation factor IF-2	654	59.97	60.00
infB_R	GGAGGTGCAAACAACACGTC			59.97	55.00
