# Supplementary material for: Multilocus sequence typing, biochemical and antibiotic resistance characterizations reveal diversity of North American strains of the honey bee pathogen Paenibacillus larvae
Source: PLoS One. 2017 May 3;12(5):e0176831. doi: 10.1371/journal.pone.0176831 (PMC5415181; doi:10.1371/journal.pone.0176831)
Supplement: S3 Table — (PDF) [file pone.0176831.s003.pdf]

S3 Table. Biochemistry profiles of *Paenibacillus larvae* by using API® tests.

Biochemistry profile — Carbohydrate metabolism

Isolates	Glycerol	D-ribose	D-xylose	L-xylose	D-glucose	D-fructose	D-manose	D-manitol	Methyl-αD-Glucopyranoside	N-acetylGlucosamine	ESCulin ferric citrate	Salicin	D-maltose	D-Lactose	D-Saccharose	D-trehalose	AmiDon	Glycogen	Xylitol	D-turanose	D-tagatose	potassium 2-KetoGluconate	potassium 5-KetoGluconate
PL-1	+	+			+	+				+	+					+					+	+	
PL-2	+	+			+		+			+	+					+						+	+
PL-3	+	+			+	+	+			+	+					+					+	+	
PL-4	+	+			+	+	+			+	+					+					+	+	
PL-5	+	+			+		+			+	+					+					+	+	+
PL-6	+	+			+		+			+	+					+						+	+
PL-7	+	+			+	+				+	+					+					+	+	
PL-8	+	+			+	+	+			+	+					+					+	+	
PL-9	+	+			+		+			+	+					+						+	+
PL-10	+	+			+					+	+					+					+	+	+
PL-11	+	+			+		+			+	+					+					+	+	+
PL-12	+	+			+					+	+					+					+	+	+
PL-13											+											+	+
PL-14	+	+			+		+			+	+			+	+	+					+	+	+
PL-15	+				+					+	+					+						+	+
PL-16	+	+			+					+	+					+					+	+	+
PL-17	+	+			+		+			+	+					+					+	+	+
PL-18	+	+			+	+	+			+	+					+					+	+	
PL-19	+	+			+	+	+			+	+					+					+	+	+
PL-20	+	+			+					+	+					+					+	+	+
PL-21	+	+			+					+	+					+					+	+	
PL-22	+	+			+	+	+			+	+					+					+	+	+
PL-23	+	+			+					+	+					+					+	+	
PL-24	+	+			+					+	+					+					+	+	+
PL-25	+	+			+					+	+					+					+	+	
PL-26	+	+			+		+			+	+					+					+	+	+
PL-27	+	+			+					+	+		+			+						+	+
PL-28	+	+			+					+	+					+					+	+	+
PL-29	+	+			+	+				+	+					+					+	+	
PL-30	+	+			+					+	+					+					+	+	
PL-31	+	+			+					+	+					+						+	+
PL-32	+	+			+	+	+			+	+	+				+						+	
PL-33	+	+			+		+			+	+					+			+		+	+	+
ATCC9545	+	+			+	+	+			+	+					+					+	+	
ATCC49843	+	+			+		+	+	+	+	+		+			+					+	+	
233/00	+	+	+	+	+			+		+	+					+					+	+	+
LMG16248	+	+			+		+	+	+	+	+		+			+					+	+	
LMG16252		+			+	+	+	+	+	+	+		+			+	+	+		+	+	+	+

Biochemistry profile — Enzymatic activity

Isolates	Alkaline phosphatase	Esterase (C 4)	Esterase Lipase (C 8)	Lipase (C 14)	Leucine arylamidase	Valine arylamidase	Cystine arylamidase	Trypsin	α-chymotrypsin	Acid phosphatase	Naphthol-AS-BI-phosphohydrolase	α-galactosidase	ß-Galactosidase	α-Glucosidase	ß-Glucosidase	N-acetyl-ß-Glucosaminidase	α-Mannosidase	α-Fucosidase
PL-1	+	+	+						+	+	+	+			+	+	+	
PL-2	+	+	+							+	+					+		
PL-3	+	+	+						+	+	+					+		
PL-4	+	+	+						+	+	+					+		
PL-5	+	+	+		+	+	+	+		+	+					+		
PL-6	+	+	+		+	+	+			+	+					+		
PL-7	+	+	+						+	+	+				+	+	+	
PL-8	+	+	+						+	+	+				+	+	+	
PL-9	+	+	+	+			+			+	+					+		
PL-10	+	+	+	+			+			+	+					+		
PL-11	+	+	+	+			+			+	+					+		
PL-12	+	+	+							+	+					+		
PL-13	+	+	+							+	+					+		
PL-14	+	+	+				+			+	+					+		
PL-15	+	+	+				+			+	+					+		
PL-16	+	+	+						+	+	+	+			+	+		
PL-17	+	+	+	+			+			+	+					+		
PL-18	+	+	+						+	+	+					+		
PL-19	+	+	+							+	+			+		+		
PL-20	+	+	+						+	+	+					+		
PL-21	+	+	+						+	+	+					+		
PL-22	+	+	+						+	+	+				+	+	+	
PL-23	+	+	+				+			+	+					+		
PL-24	+	+	+	+	+	+		+		+	+	+	+	+		+		+
PL-25	+	+	+							+	+					+		
PL-26	+	+	+	+	+	+		+		+	+	+	+			+	+	+
PL-27	+	+	+	+	+	+	+			+	+	+				+		
PL-28	+	+	+						+	+	+				+	+	+	
PL-29	+	+	+						+	+	+					+		
PL-30	+	+	+						+	+	+					+		
PL-31	+	+	+	+			+			+	+					+		
PL-32	+	+	+						+	+	+					+		
PL-33	+	+	+		+	+		+		+	+	+		+		+		
ATCC9545	+	+	+						+	+	+					+		
ATCC49843	+	+	+						+	+	+					+		
233/00		+	+							+	+			+		+		
LMG16248	+	+	+						+	+	+				+	+	+	+
LMG16252	+	+	+		+	+	+	+		+	+					+		

API testing systems

API® 50 CH

API® ZYM
