# Supplementary figures and images for: Multilocus sequence typing, biochemical and antibiotic resistance characterizations reveal diversity of North American strains of the honey bee pathogen Paenibacillus larvae
Source: PLoS One. 2017 May 3;12(5):e0176831. doi: 10.1371/journal.pone.0176831 (PMC5415181; doi:10.1371/journal.pone.0176831)

**S1 Fig. ERIC PCR genotyping of 38 *Paenibacillus larvae* isolates.**

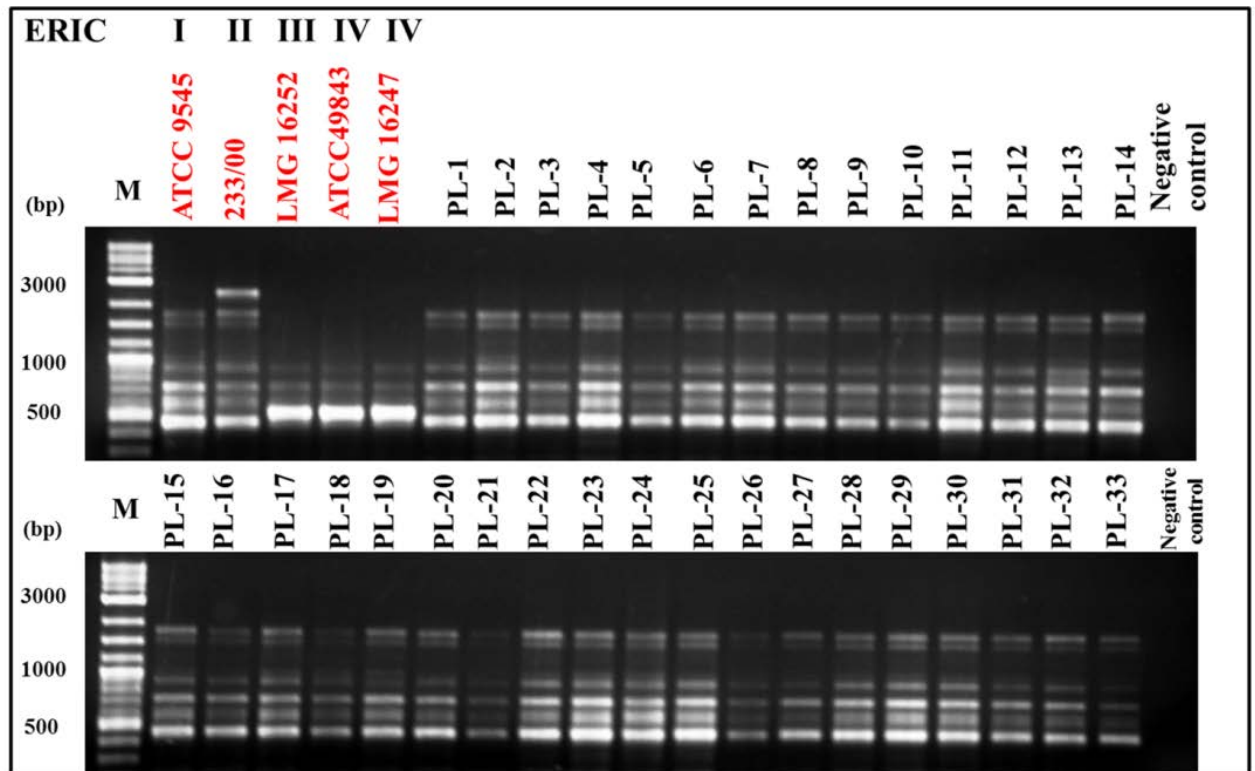

Supplement: S1 Fig — (PDF) [file pone.0176831.s004.pdf]

S2 Fig. BOX PCR genotyping of 38 *Paenibacillus larvae* isolates.

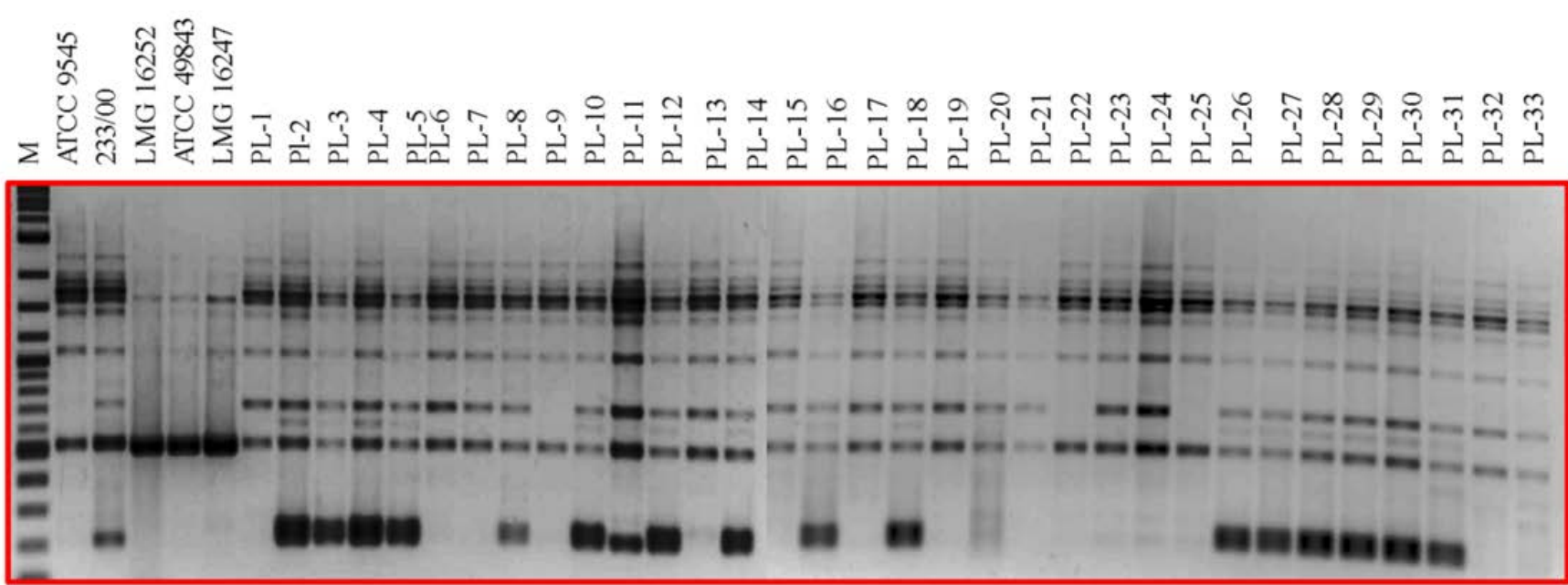

Supplement: S2 Fig — (PDF) [file pone.0176831.s005.pdf]
