# Supplementary material for: Multilocus sequence typing, biochemical and antibiotic resistance characterizations reveal diversity of North American strains of the honey bee pathogen Paenibacillus larvae
Source: PLoS One. 2017 May 3;12(5):e0176831. doi: 10.1371/journal.pone.0176831 (PMC5415181; doi:10.1371/journal.pone.0176831)

S3 Fig. Presentation of all amplified products by using designed MLST primers. In red boxes are seven loci used in the studies.

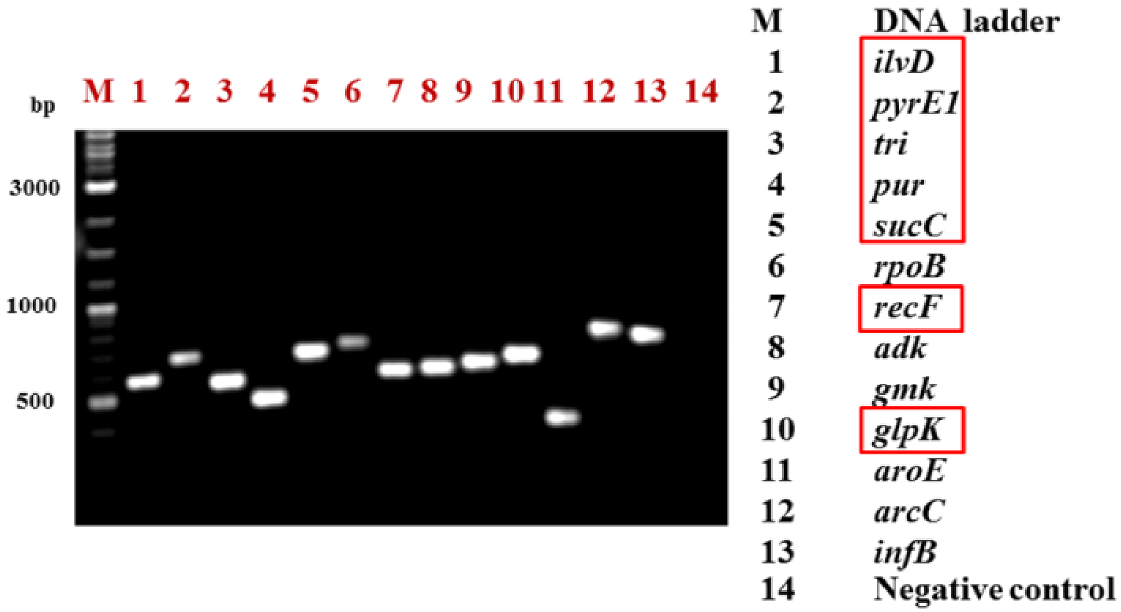

Supplement: S3 Fig — In red boxes are seven loci used in the studies. (PDF) [file pone.0176831.s006.pdf]
